# Editorial: Insights in veterinary regenerative medicine: 2023

**DOI:** 10.3389/fvets.2024.1483576

**Published:** 2024-09-09

**Authors:** Debbie J. Guest, Scott J. Roberts

**Affiliations:** ^1^Department of Clinical Science and Services, Royal Veterinary College, Hatfield, United Kingdom; ^2^Department of Comparative Biomedical Sciences, The Royal Veterinary College, London, United Kingdom

**Keywords:** regenerative medicine, veterinary, skin, tendon, cartilage

The veterinary regenerative medicine field continues to grow as demand for new treatments for chronic and degenerative conditions increases. In this Research Topic we aimed to gather new insights, novel developments, latest discoveries, and recent advances in the field. In addition, we sought to identify Research Topics on the current challenges and future perspectives that could move the field forward.

Although there is a large body of *in vitro* work that is being performed in the field of regenerative medicine, the articles submitted to this Research Topic all performed *in vivo* studies. Faydaver et al. used mouse models; the other three studies all used large animals, with Iacopetti et al. and Peláez-Gorrea et al. utilizing sheep and Andersen et al. using horses. This likely reflects the advantages of using large animal models to aid clinical translation (1). However, large animal studies are expensive and therefore often involve small numbers. This limitation was highlighted by the authors who acknowledged their small sample sizes and/or the need to induce injury in multiple limbs. The use of laboratory mice enables much higher numbers of animals to be used due to both cost and space factors, but determining the effects of a therapy on tissue regeneration over time can still require very large animal numbers to enable collection of the tissue at multiple time points. Faydaver et al. tried to address this issue by predicting the recovery of tendon structure after injury in mice by utilizing high-resolution spatiotemporal data on activity patterns obtained through continuous, non-invasive home-cage monitoring. The development of such techniques could significantly refine pre-clinical studies into regenerative therapies and lead to reductions in animal numbers.

The studies in this Research Topic investigated injury and healing of skin (Iacopetti et al.), cartilage (Peláez-Gorrea et al.; Andersen et al.), and tendons (Faydaver et al.). Veterinary stem cell clinical trials have been most commonly performed for musculoskeletal disorders (2), reflecting the economic and welfare burden of these conditions in veterinary species. All of the models in this Research Topic used experimentally induced, rather than naturally occurring, injuries. This reduces variability in the model, but as Andersen et al. described, it is difficult to ascertain how well such models mimic spontaneous disease/injury.

Three of the studies were testing biological products: Iacopetti et al. used either autologous platelet-rich plasma (PRP) or autologous PRP plus allogenic mesenchymal stromal cells (MSCs) in their model of skin healing; Peláez-Gorrea et al. used autologous chondral platelet-rich plasma matrix in cartilage repair; and Andersen et al. used allogeneic MSCs selected for the marker integrin α10 to determine their effect on microRNA expression in an osteoarthritis model. The debate on the safety and efficacy, particularly following repeat administration, on the use of allogeneic MSCs continues (3). However, the clinical application of both allogeneic (4) and xenogenic MSCs (5) is rapidly expanding. PRP remains a popular biological therapeutic in companion animals due to its relatively low expense and ease of preparation. However, there remains considerable variability in methods of preparation, and systematic reviews in human applications have produced mixed results (6, 7). The studies submitted to this Research Topic would suggest that research into PRP and allogeneic cells is going to continue amid a rising demand for cost-effective, off-the-shelf products.

The studies submitted to this Research Topic measured outputs ranging from microRNA analysis (Andersen et al.) and histology and gene expression (Iacopetti et al.) to activity (Faydaver et al.) and clinical evaluation (Iacopetti et al.; Peláez-Gorrea et al.). This demonstrates the complexity of determining successful tissue regeneration. Furthermore, it also highlights that current high-resolution analysis techniques used in human research, such as special transcriptomics and epigenome profiling, are not yet commonplace in veterinary regenerative medicine research. It is our hope that this is due to technical challenges and not limited funding for veterinary studies.

In summary, although this was a brief Research Topic, the collection of studies has highlighted ongoing research in biological products, the use of experimentally induced animal models, and possible refinements that could be made for future studies.

## References

[1] Ribitsch I Baptista PM Lange-Consiglio A Melotti L Patruno M Jenner F. Large animal models in regenerative medicine and tissue engineering: to do or not to do. Front Bioeng Biotechnol. (2020) 8:972. 10.3389/fbioe.2020.00972 32903631 PMC7438731

[2] Williams ZJ Pezzanite LM Chow L Rockow M Dow SW. Evaluation of stem-cell therapies in companion animal disease models: a concise review (2015–2023). Stem Cells. (2024) 2024:sxae034. 10.1093/stmcls/sxae034 38795363 PMC13032169

[3] Cequier A Vázquez FJ Vitoria A Bernad E Fuente S Serrano MB. The systemic cellular immune response against allogeneic mesenchymal stem cells is influenced by inflammation, differentiation and MHC compatibility: *in vivo* study in the horse. Front Vet Sci. (2024) 11:1391872. 10.3389/fvets.2024.1391872 38957800 PMC11217187

[4] Carlier S Depuydt E Van Hecke L Martens A Saunders J Spaas JH. Safety assessment of equine allogeneic tenogenic primed mesenchymal stem cells in horses with naturally occurring tendon and ligament injuries. Front Vet Sci. (2024) 11:1282697. 10.3389/fvets.2024.1282697 38468694 PMC10925754

[5] Punzón E Salgüero R Totusaus X Mesa-Sánchez C Badiella L García-Castillo M. Equine umbilical cord mesenchymal stem cells demonstrate safety and efficacy in the treatment of canine osteoarthritis: a randomized placebo-controlled trial. J Am Vet Med Assoc. (2022) 260:1947–55. 10.2460/javma.22.06.0237 36198051

[6] Boksh K Elbashir M Thomas O Divall P Mangwani J. Platelet-Rich Plasma in acute Achilles tendon ruptures: a systematic review and meta-analysis. Foot. (2022) 53:101923. 10.1016/j.foot.2022.101923 36037774

[7] Wang C Fan H Li Y Yun Z Zhang Z Zhu Q. Effectiveness of platelet-rich plasma injections for the treatment of acute Achilles tendon rupture: a systematic review and meta-analysis. Medicine. (2021) 100:e27526. 10.1097/MD.0000000000027526 34731144 PMC8519239

